# Association between Severity of Freezing of Gait and Turning Characteristics in People with Parkinson’s Disease

**DOI:** 10.3390/ijerph191912131

**Published:** 2022-09-25

**Authors:** Hyejin Choi, Changhong Youm, Hwayoung Park, Bohyun Kim, Sang-Myung Cheon, Myeounggon Lee

**Affiliations:** 1 Department of Health Sciences, The Graduate School, Dong-A University, Saha-gu, Busan 49315, Korea; 2 Department of Health Care and Science, Dong-A University, Saha-gu, Busan 49315, Korea; 3 Department of Neurology, School of Medicine, Dong-A University, Seo-gu, Busan 49201, Korea; 4 Interdisciplinary Consortium on Advanced Motion Performance (iCAMP), Michael E. DeBakey Department of Surgery, Baylor College of Medicine, Houston, TX 77030, USA

**Keywords:** Parkinson’s disease, freezing of gait, gait, motor deficits, kinematics

## Abstract

For people with Parkinson’s disease (PD) with freezing of gait (FOG) (freezers), symptoms mainly exhibit as unilateral motor impairments that may cause difficulty during postural transitions such as turning during daily activities. We investigated the turning characteristics that distinguished freezers among people with PD and analyzed the association between the New Freezing of Gait Questionnaire (NFOGQ) scores and the gait characteristics according to the turning direction for the affected limbs of freezers. The study recruited 57 people with PD (27 freezers, 30 non-freezers). All experiments measured the maximum 180° turning task with the “Off” medication state. Results revealed that the outer ankle range of motion in the direction of the inner step of the more affected limb (IMA) was identified to distinguish freezers and non-freezers (R_N_^2^ = 0.735). In addition, higher NFOGQ scores were associated with a more significant anteroposterior root mean square distance of the center of mass in the IMA direction and a greater inner stance phase in the outer step of the more affected limb (OMA) direction; explanatory power was 50.1%. Assessing the maximum speed and turning direction is useful for evaluating the differences in turning characteristics between freezers and non-freezers, which can help define freezers more accurately.

## 1. Introduction

Freezing of gait (FOG) is defined as a symptomatic episodic absence of forward progression or marked reduction in step length, despite the intention to walk [1], and approximately 80% of people with Parkinson’s disease (PD) experience FOG [1,2]. FOG symptoms are mainly caused by turning, changing directions, multi-tasking, passing through narrow passages, moving when pressed for time, and approaching destinations [3]. Thus, FOG symptoms not only affect the daily life and movements of people with PD but are also related to an increased risk of falls [2,3].

The New Freezing of Gait Questionnaire (NFOGQ) checks for the presence or absence of FOG and objectively evaluates the severity [4]. In addition, there are various methods for evaluating the disease severity of people with PD, such as the Unified Parkinson’s Disease Rating Scale (UPDRS) and the modified Hoehn and Yahr scale [5]. However, a major limitation of using the questionnaire is the subjective evaluation [6]. FOG is related to decreased bilateral coordination of periodic limb movement, and increased severity of FOG in people with PD with FOG (freezers) correlates with increased gait asymmetry [7]. However, these studies used the straight gait task and were merely the results of a simple correlation analysis. People with PD have difficulty turning due to the unilateral predominance of disease symptoms [8]. Recent studies have examined in-depth the turning of people with PD through spatiotemporal and kinematic variables using three-dimensional (3D) motion capture systems, a current gold standard. The results have been compared between freezers and non-freezers [9,10,11]. In particular, several studies have analyzed turning characteristics considering the directions toward the inner step of the more affected limb (IMA) and the outer step of the more affected limb (OMA) defined as the disease-dominant side [9,12]. Mainly, freezers have increased cadence [12] and decreased inner step length [9] when turning toward the more affected limb. However, few studies have analyzed the relationship between turning characteristics considering the direction and asymmetric disease symptoms of freezers [9,12]. In addition, increased attention and effort to impress the examiner during assessment in the laboratory or a clinical visit may improve one’s gait performance [2]. Freezers have greater difficulty in challenging tasks such as gait and turning with the condition of maximum speed [10]. Therefore, we used a challenging 180° turning task at the maximum speed that can further affect cognition and physical performance [10], and a body marker-based movement analysis system that can analyze in-depth the full-body turning characteristics according to the direction of the more and less affected limbs. Such an approach may help classify the turning characteristics of freezers among people with PD, determine motor severity, and assist in fall prevention.

Freezers have higher cadence, increased step time variability, and disordered bilateral coordination during turning, deficits that correlate with a greater number of FOG episodes [13,14]. Furthermore, Park et al. [15] identified the association between turning characteristics and NFOGQ scores including clinical characteristics demonstrating that increased disease severity in freezers was associated with motor deficits such as stepping inhibition and loss of automaticity during repeated weight locomotion during turning. In addition, provoking FOG or gait instability may occur more frequently during the “end” stage, which is the fourth turning quadrant of the 180° turning task [10,16]. However, the relationship between turning characteristics and the severity of FOG remains unclear [4,17,18]. Therefore, it is necessary to establish the relationship between turning characteristics and disease severity by identifying the combination of turning characteristics that may pathologically and sensitively determine degeneration levels of freezers.

This study aimed to distinguish between freezers and non-freezers based on 180° turning characteristics and to investigate the associations between NFOGQ scores, which determine the presence and severity of the FOG and 180° turning characteristics at the maximum speed. According to our hypotheses, the 180° turning characteristics based on a full-body kinematic analysis may distinguish freezers from non-freezers and have a strong association with the NFOGQ score.

## 2. Materials and Methods

### 2.1. Participants

A total of 125 people with PD were recruited at the Department of Neurology of a local medical center. People who did not meet the inclusion criteria and refused to participate were excluded, and 73 people with PD were allocated to the groups (freezers and non-freezers). The drop-out criteria were as follows: (1) those who withdrew their informed consent; (2) those who could not attend the entire experiment due to severe symptoms; (3) and those who showed FOG symptoms in the turning phase. Therefore, this study conducted the final analysis with 57 people with PD (27 freezers, 30 non-freezers). Their condition was diagnosed by a neurologist according to the UK Parkinson’s Disease Society Brain Bank criteria [19]. Inclusion criteria were as follows: (1) patients aged 50–85 years; (2) those that could walk independently without aids; (3) those receiving stable treatment with antiparkinsonian medications; (4) those having a modified Hoehn and Yahr stage 2–3 [5]; (5) those with a Mini-Mental State Examination (MMSE) > 24 [20]; and (6) those classified as freezers or non-freezers (assessed with or without FOG by an NFOGQ score of >3 and ≤3, respectively) [4]. Exclusion criteria were as follows: (1) patients with a medical diagnosis of any other neurological, musculoskeletal, cardiovascular, or vestibular disorder; (2) those that had undergone previous brain surgeries; and (3) those that required assistive devices for moving.

One participant had a turning task-induced FOG episode with severe symptoms difficult to analyze. Therefore, data for this participant was excluded.

**Figure 1:**
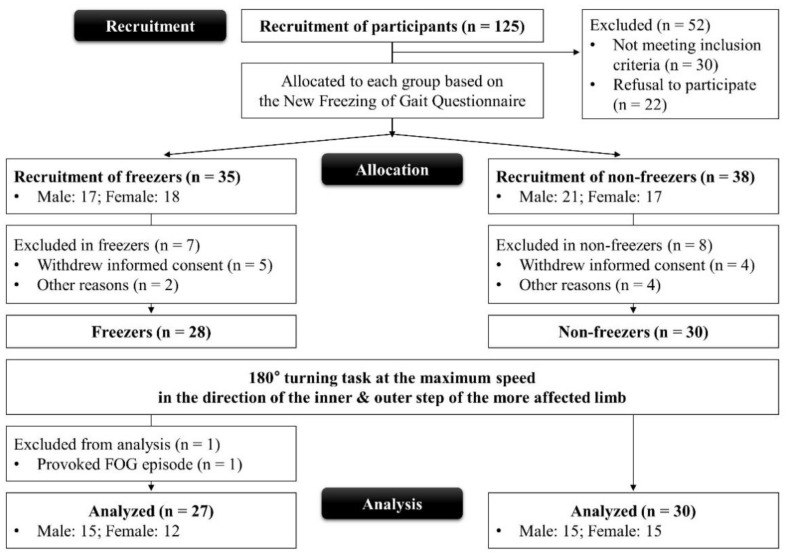
Study flow chart. FOG, freezing of gait.

The study flow chart is shown in Figure 1, and the physical and clinical characteristics of both groups are provided in Table 1. All experiments were performed in accordance with relevant guidelines and regulations. The experimental protocols were approved by the Institutional Review Board (IRB) of Dong-A University Medical Center (IRB number: DAUHIRB-17-033). Written informed consent was obtained from all participants before they participated in this study.

### 2.2. Experiment Procedures

Participants performed two sessions. In the first session, the participants completed the informed consent form and were assessed using the UPDRS, modified Hoehn and Yahr scale, NFOGQ, and MMSE (Table 1). In the second session, the 180° turning task was performed at the maximum speed, which was defined as the fastest speed at which the participants chose to perform the tasks. All experiments were performed in the “Off” medication state, with medication withdrawn at least 12 h before the measurements.

All participants wore fitted T-shirts and shorts for convenience, and the turning task was performed barefoot. First, body height, body weight, shoulder offset, elbow width, wrist width, hand thickness, leg length, knee width, and ankle width measurements were obtained to estimate the joint kinematics data. Then, 39 reflective markers in the shape of spheres (14 mm each) were placed according to the Plug-in Gait full-body model (Vicon Motion Systems Ltd., Oxford Metrics, Oxford UK) [21].

**Figure 2:**
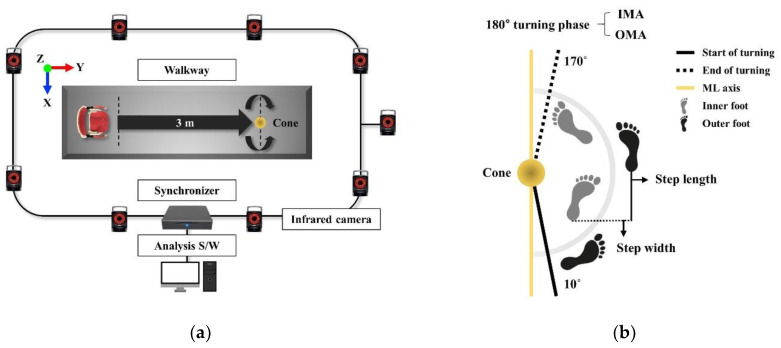
Schematic representation of experimental setup and analysis phase. (**a**) Placement of experimental equipment; X, Y, and Z represent the global coordinate system; (**b**) The analysis phase of the 180° turning task at the maximum speed includes the definition of step width and step length; IMA, inner step of the more affected limb; OMA, outer step of the more affected limb; ML, mediolateral.

The participants warmed up by walking and stretching for approximately 5 min before the experiment started. Then, the participants were instructed to practice the 180° turning task at the maximum speed with 3 to 5 trials, and the measurements were conducted after approximately 5 min of rest. After the initial rest period, the participants were instructed to sit on a chair. At the beginning signal using the bell, the participants were asked to rise from sitting on a standard chair, walk 3 m, turn around a cone, walk back to the chair, and sit down at maximum speed. The 180° turning task at the maximum speed used in this study was modified from the timed up and go test (Figure 2a) [11].

The 180° turning task at maximum speed was performed in the IMA and OMA directions, three times each in random order. The direction of the more affected limb was defined as the asymmetry level in the motor symptoms of the participants using the items that individually evaluated the physical function of the UPDRS, and the pathological symptoms were more dominantly observed [22]. A neurologist conducted this process. The participants successfully performed the 180° turning tasks three times randomly depending on each direction, with 30 s of rest between trials.

### 2.3. Data Acquisition and Analyses

The 3D motion data were collected using nine infrared cameras (Vicon MX-T10, Oxford Metrics), the synchronizer (Giganet, Oxford Metrics), and the Vicon Nexus software (version 2.10.3, Oxford Metrics) (Figure 2a). For the global coordinate system, the position of the left posterior ground of the participant was set as the origin, and the positive *X*-axis, *Y*-axis, and *Z*-axis faced the right, anteriorly, and superiorly, respectively. In addition, the *Z*-axis was defined as the cross-product between the *X*-axis and *Y*-axis. The collected data were sampled at 100 Hz and filtered using a fourth-order Butterworth low-pass filter with a 10 Hz cutoff frequency [9,15].

The analysis phase during the 180° turning task included—the “start event” set to the point where the angle between the anterior superior iliac spine (ASIS) vector (defined as the distance between the left ASIS and right ASIS) and the “axis vector” (defined as the X-axis vector of the global coordinate system) exceeded 10° on the horizontal plane; the ending event was set to the point where the two vectors reached 170° (Figure 2b) [9].

**Figure 3:**
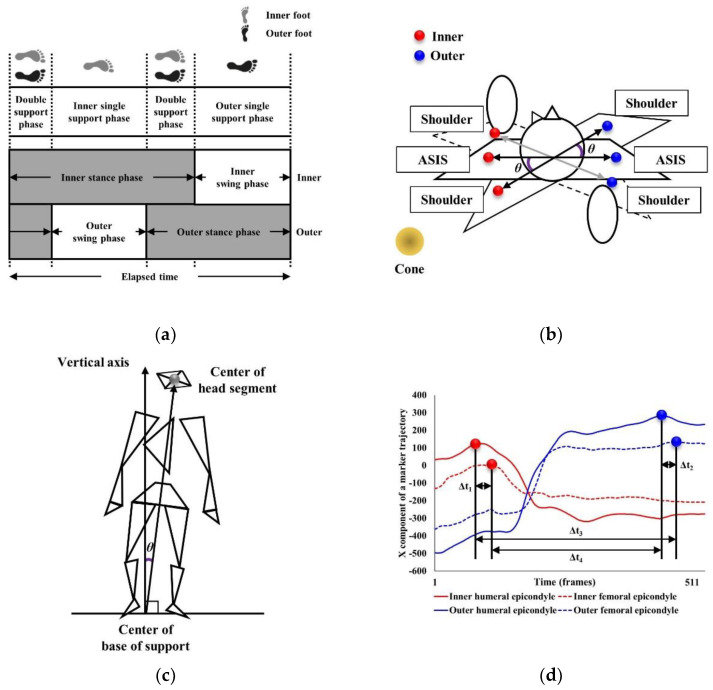
Analysis variables. (**a**) The definition of inner and outer double and single support and stance phases; (**b**) The definition of the maximum anti-phase; (**c**) The definition of the incline angle; (**d**) The definition of the inner and outer ipsilateral and contralateral temporal coordination parameters of the upper and lower limbs; ASIS, anterior superior iliac spine.

The analysis variables, spatiotemporal, kinematic, and center of mass (COM) parameters during the 180° turning tasks at maximum speed were calculated using MATLAB R2020b (MathWorks, Natick, MA, USA). An average of three trials was used in the analysis. The definitions of the analysis variables are presented in Table 2, Figure 2 and Figure 3.

### 2.4. Statistical Analyses

At study commencement, we performed a priori power analysis. This analysis showed that a minimum sample size of 56 participants was required to achieve a statistical significance of 0.05 with a power of 80% at an overall effect size of 0.20 when set to 2 for the number of groups and 4 in the number of measurements. Furthermore, considering the rate of dropouts that may occur during the experiment, the number of participants was set as 33 freezers and 33 non-freezers [23,24]. The sample size calculation was performed using G-power (version 3.1.0).

All statistical analyses were performed using SPSS 21.0 (IBM Corp., Armonk, NY, USA), and the statistical significance level was set at 0.05. The Shapiro–Wilk test was used to assess data normality. Fisher’s exact test and Mann-Whitney *U* test (for non-normally distributed data) or independent *t*-test (for normally distributed data) were conducted to assess the differences in physical and clinical characteristics between freezers and non-freezers. In addition, a two-way analysis of variance with repeated measures was performed to investigate the main effects and interactions of freezers and non-freezers and between the turning directions (IMA and OMA directions) of the 180° turning tasks at the maximum speed. Finally, the post hoc test was conducted using the analysis of covariance (ANCOVA) or the Mann-Whitney *U* test between freezers and non-freezers. The selected covariates were age, sex, height, and body mass index (BMI) in ANCOVA to minimize the influence of these factors affecting the participants’ demographic characteristics. Additionally, the effect size was calculated using Cohen’s d. This study aimed to identify variables that can distinguish groups, so we focused on the results comparing the groups.

After Z standardization was performed with the turning characteristics that showed differences between groups, a stepwise binary logistic regression analysis was performed to identify classifier variables for distinguishing freezers and non-freezers. The covariates and turning characteristics set for the first and second blocks were age, sex, height, BMI, and differences between freezers and non-freezers (disease duration, levodopa equivalent dose, Hoehn and Yahr scale, UPDRS total scores, and UPDRS Part II scores). The classifier variable was expressed as an odds ratio with a 95% confidence interval (CI). In addition, the area under the curve (AUC) was calculated through the turning characteristic’s receiver operating characteristic (ROC) curve analysis of the turning characteristic to identify the classification accuracy of freezers and non-freezers. Finally, Youden’s index was used to analyze the optimal cutoff value of the variable that distinguished the freezers from non-freezers. AUC > 0.9 indicates high accuracy, whereas AUCs of 0.7–0.9 and 0.5–0.7 indicate moderate and low accuracies, respectively [25]. A partial correlation analysis was performed to examine the relationship between the NFOGQ scores, which determined the presence and severity of FOG, and all turning characteristics in freezers. Furthermore, a stepwise multivariable linear regression analysis was performed to identify the association between variables that significantly correlated with the freezers’ NFOGQ scores. Covariates and all turning characteristics were applied to the first and second blocks. The NFOGQ score was applied as the dependent variable. The selected covariates were age, sex, height, and BMI in these statistical processing to minimize the influence of factors affecting the participants’ demographic characteristics.

## 3. Results

### 3.1. Classifier Variable for Freezers and Non-Freezers

Post hoc analyses revealed an effect of groups; the differences in 180° turning characteristics freezers had significantly greater total steps (*p* = 0.031), outer double support phase (*p* = 0.036), and lower outer ankle range of motion (ROM) (*p* = 0.048) when compared with non-freezers in the IMA direction. In addition, freezers demonstrated significantly lower inner hip ROM (*p* = 0.049) than non-freezers in the OMA direction. All characteristics analyzed during the 180° turning task are summarized in Supplementary Table S1.

**Figure 4:**
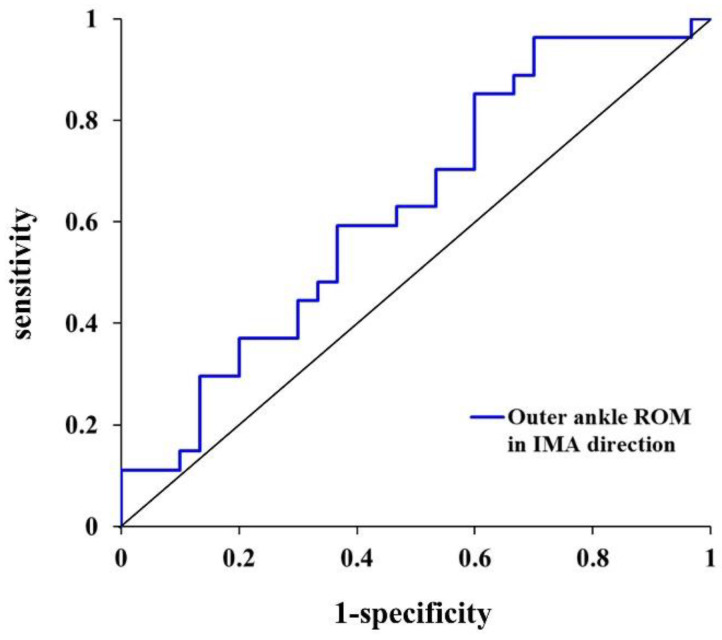
Receiver operating characteristic curve of classifier variable for freezers and non-freezers during the 180° turning task. ROM, range of motion; IMA, inner step of the more affected limb.

The results of the stepwise binary logistic regression and ROC curve analysis (Figure 4 and Table 3) showed that the variable that best distinguished freezers and non-freezers was the outer ankle ROM (cutoff value: 31.0°, AUC: 0.628, *p* = 0.026, sensitivity: 0.593, specificity: 0.600) in the IMA direction, and the explanatory power was 73.5% (R_N_^2^ = 0.735).

### 3.2. Association between NFOGQ Score and 180° Turning Characteristics in Freezers

We first assessed the correlation between NFOGQ scores and turning characteristics in freezers. The NFOGQ score showed a significant negative correlation with inner toe clearance height (r = −0.468, *p* = 0.024) and a significant positive correlation with anteroposterior (AP) root mean square (RMS) distance of the COM (r = 0.436, *p* = 0.037) in the IMA direction. In the OMA direction, the NFOGQ score had a significant positive correlation with inner stance phase (r = 0.502, *p* = 0.015). The variables in the results of the correlation analysis are presented in Supplementary Table S2.

**Figure 5:**
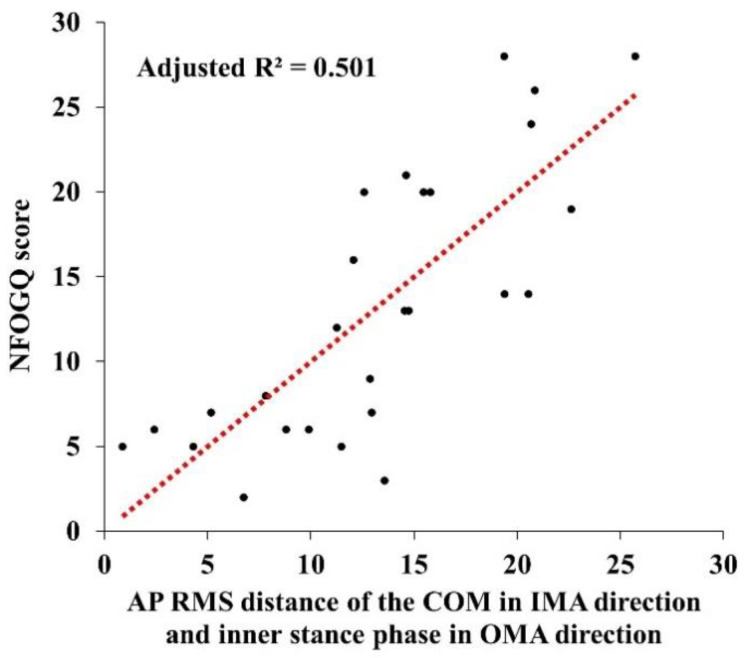
Associations between NFOGQ scores and turning characteristics for freezers during the 180° turning task. Tendency of regression line indicates positive linear correlation of corresponding correlation coefficient; NFOGQ, New Freezing of Gait Questionnaire; AP, anteroposterior; RMS, root mean square; COM, center of mass; IMA, inner step of the more affected limb; OMA, outer step of the more affected limb.

Then, we further analyzed the associations between NFOGQ scores and turning characteristics using a stepwise multivariable linear regression model after adjusting for age, sex, height, and BMI. The results for freezers showed that the higher NFOGQ score was associated with greater AP RMS distance of the COM (*p* = 0.045) in the IMA direction and greater inner stance phase (*p* = 0.018) in the OMA direction (Figure 5 and Table 4). Explanatory power was 50.1% (adjusted R^2^ = 0.501). This is the linear regression equation:

Y (NFOGQ score) = 160.710 + 61.272 X_1_ (AP RMS distance of the COM in the IMA direction) + 1.027 X_2_ (inner stance phase in the OMA direction) (1)

## 4. Discussion

Our study revealed that freezers had significantly greater total steps, outer double support phase, lower outer ankle ROM in the IMA direction, and lower inner hip ROM in the OMA direction than non-freezers. The stepwise binary logistic regression results demonstrated that the outer ankle ROM distinguished between freezers and non-freezers in the IMA direction. Additionally, the NFOGQ score showed a significant negative correlation with inner toe clearance height and a positive correlation with AP RMS distance of the COM in the IMA direction. In the OMA direction, the NFOGQ score had a significant positive correlation with the inner stance phase. Furthermore, AP RMS distance of the COM in the IMA direction and inner stance phase in the OMA direction were associated with the NFOGQ scores of freezers. These results demonstrated our hypothesis that 180° turning characteristics may have enabled the distinction between freezers and non-freezers, and their association with the NFOGQ scores.

### 4.1. Classifier Variables According to 180° Turning Characteristics for Freezers and Non-Freezers

Freezers have more significant difficulties in turning due to the need for modification of movement patterns that require cognitive and executive functions in the frontal lobe, more inter-limb coordination, posture control, and coupling between posture and gait and turning tasks, including the maximum speed, which can give them incredible difficulty [10]. Moreover, freezers may experience FOG during turning due to abnormal control outputs from the central pattern generators (CPG) in the spinal cord, a neural network responsible for goal-directed motor output important for locomotion adaptation, and disruption of the supraspinal control cues to the CPG [26].

Our results were consistent with recent studies that suggested freezers significantly increase total steps when turning as fast as possible in the “Off” state of medication [1,9]. These results may be associated with increased posture tone, axial rigidity, and loss of intersegmental flexibility during the turning of freezers [27]. In addition, reduction in ankle ROM may affect not only the difficulty in initiating turning, and deterioration of gait and balance asymmetry, but also step length and speed, eventually increasing the total steps required to complete turning [2,10,28]. Wang et al. [28] associated poorer ankle proprioceptive acuity with increased cadence and decreased step length, suggesting that shuffling gait observed in freezers may be related to ankle proprioceptive impairment.

The basal ganglia involved in automatic movement serve to transmit signals step by step to the supplementary motor area that regulates gait control, and damaged neural output from the basal ganglia to the supplementary motor area may degrade the ability to control both sides for gait, causing asymmetry [7]. Consequently, these characteristics may be exacerbated in the “Off” medication state, and gait characteristics such as shuffling and walking with a short step length while dragging the foot on the ground may increase [3]. In addition, freezers with already compromised dynamic stability exhibit more cautious movements during turning and may use more restricted postural strategies to promote effective turning from dopamine-depleted states that may affect the control of automatized movements [15]. In particular, they use compensatory and adaptive strategies, such as increasing the total steps to provide more postural stability and to prevent falls during turning due to more significant impairment of cognitive, executive, and attentional resources compared with non-freezers [15,27].

In addition, our study analyzed turning direction. Interestingly, the stepwise binary logistic regression analysis identified the variables capable of distinguishing between freezers and non-freezers in the IMA direction. For instance, freezers showed significantly greater ankle ROM than non-freezers in the IMA direction. Although the mechanism for unilateral motor symptoms in people with PD has not yet been identified, the difference in absorption of dopamine by striatal dopamine receptors (D1 and D2) between the caudate and putamen nuclei—the areas responsible for motor performance control [19]—not only interferes with the activity of the basal ganglia circuit [29] but also inhibits frontocortical area activity and causes asymmetric neurodegeneration [8].

These asymmetric characteristics of freezers affect inter-limb coordination and decrease the ability to posture control during movement due to posture instability in daily life [3]. Considering the generalized turning characteristics, the increased double support phase of the outer lower limb during the turning may be a general result. In addition, previous studies reported that freezers increased gait asymmetry regardless of the affected limb by the unilateral motor symptoms [14,22]. However, other previous studies reported that freezers had more difficulty in turning tasks due to increased asymmetry related to posture control and movement when turning in the IMA direction [9,30]. Similarly, as a result of our study, the increase in the double support phase of the outer lower limb when turning in the IMA direction of freezers may be a strategy to compensate through the outer lower limb due to the decrease in the function of the more affected limb [9]. Therefore, increased rigidity during turning in freezers caused by reduced automatic and spontaneous motor ability may result in an increased risk of falling compared to non-freezers [31].

### 4.2. 180° Turning Characteristics Associated with NFOGQ Score in Freezers

We showed that several 180° turning characteristics were related to the NFOGQ scores in freezers, and the results of the linear regression analysis model were able to further establish the association through a robust explanatory power of 50.1%. In previous studies, as NFOGQ scores in freezers increased, variables with a relationship between gait characteristics were reported, such as reduced step length, decreased mediolateral (ML) center of pressure, and increased gait asymmetry [7,18]. However, these results were derived from step initiation, and treadmill walking tasks.

Furthermore, freezers require a more dynamic balance than straight gait due to increased activities of the frontal cortex and basal ganglia circuits during turning, and FOG symptoms may occur due to this increased activity [17]. Our result found that the AP RMS distance of the COM was related to the NFOGQ score. AP RMS distance refers to the degree of AP tilting using the trajectory on the horizontal plane of COM in the radius of turning. A previous study reported that freezers increased the AP RMS distance of the COM during 360° and 540° turning in the IMA direction at the maximum speed compared to that in the OMA direction [9]. These results suggest that freezers’ degradation in weight locomotion ability due to increased AP postural instability when turning in the IMA direction may be related to the severity of FOG [16]. In addition, our results indicated an increased inner stance phase when turning in the OMA direction, relying on the lower limb, which is less affected. Ultimately, freezers may exhibit turning characteristics such as asymmetric gait due to reduced ability of bilateral movement coordination [7].

Mancini et al. [32] reported that as FOG severity increases in freezers, the ability to coordinate natural movement and normal gait patterns might be impaired along with increased FOG rate and asymmetric steps during turning. These results suggest that FOG symptoms are related to the degeneration of the spinal cord pattern generator rather than the frontal cortex, including the motor cortex and supplementary motor area [32]. Therefore, the results of our study on the association between NFOGQ score and turning characteristics of the direction according to the influence of unilateral motor symptoms within freezers may be helpful for early diagnosis and prediction studies according to the severity of FOG. In addition, a recent review on FOG detection and prediction using wearable sensors reported that far too few or simple features may negatively impact classification performance [33]. Future analyses that take into account the relationship between turning characteristics employed in this study and the NFOGQ scores of freezers in a clinical-based setting may improve the accuracy of FOG prediction models with a set of optimal features.

### 4.3. Clinical Implications and Limitations

Our study has several important clinical implications. The main results of this study not only distinguished the turning characteristics of freezers among people with PD but also revealed the association between the NFOGQ scores and the gait characteristics based on the turning direction of the affected limbs of freezers. In addition, we found meaningful variables by considering clinical characteristics such as disease duration, levodopa equivalent dose, Hoehn and Yahr scale, UPDRS total scores, and UPDRS Part II scores in this analysis process for distinguishing freezers and non-freezers. We suggest that these factors need to be considered in future research designs. These approaches have an expected effect on improving the diagnosis of freezers, a severe stage affecting people with PD, and could be used as basic data for intervention, injury prevention, and rehabilitation education. We recommend that all stakeholders, such as patients, caregivers, healthcare providers, and clinicians, pay attention to turn movements of daily life for rehabilitation using turning tasks of freezers.

There are several limitations to this study. First, we did not compare the difference between the “On” and “Off” medication states while evaluating the 180° turning task. Dopaminergic medications affect gait characteristics in people with PD. Second, our results showed no significant difference in UPDRS Part Ⅲ scores (*p* = 0.655) between freezers and non-freezers despite the significant difference in the UPDRS total scores. Although the UPDRS Part Ⅲ score may contribute to evaluating the functional effect of FOG, it does not reflect the overall severity of FOG [4,31]. Lastly, only one participant had FOG symptoms in the turning phase during our experimental process, although provoking FOG during turning is common in freezers [10]. Since the turning task in the laboratory environment is different from daily life, FOG symptoms may not appear in the laboratory environment [2]. In addition, increased attention during laboratory evaluation to impress the examiner may have improved their gait performance, resulting in rare FOG symptoms [2]. Understanding FOG symptoms in daily life with the wearable sensor-based assessment may be useful in evaluating the advanced motor characteristics in people with PD [33,34]. Therefore, future studies should consider using the wearable system to explore advanced characteristics in freezers.

## 5. Conclusions

This study showed a significant difference between freezers and non-freezers in a kinematic variable during the 180° turning task at maximum speed in the IMA direction. In addition, lower turning performance might indicate increased FOG severity, which had a high explanatory power of 50.1%, such as the associations between NFOGQ scores and turning characteristics. Understanding the turning characteristics regarding maximum speed and turning direction in freezers and non-freezers will be useful for evaluating differences in turning characteristics, improving disease monitoring, and future clinical evaluations. Furthermore, our study results will help to define freezers more accurately, recognize the relationship between disease progression and impaired turning patterns, and improve the accuracy of the FOG prediction model.

## Figures and Tables

**Table 1:** Physical and clinical characteristics of all participants.

Characteristics	People with PD		*p*-Value
	Freezers (n = 27)	Non-Freezers (n = 30)	
Sex (male/female)	15/12	15/15	0.792 ^a^
Age (years)	68.05 ± 5.21	68.06 ± 4.62	0.991 ^b^
Height (cm)	157.99 ± 9.30	157.32 ± 8.38	0.776 ^b^
Body weight (kg)	59.71 ± 8.86	59.44 ± 7.53	0.902 ^b^
BMI (kg/m^2^)	23.88 ± 2.61	24.03 ± 2.61	0.831 ^b^
MMSE (scores)	28.00 ± 1.78	27.27 ± 1.80	0.105 ^c^
Disease duration (years)	9.00 ± 6.67	4.24 ± 4.04	0.001 ^c^
LED (mg/day)	745.83 ± 349.89	460.00 ± 252.03	0.001 ^c^
NFOGQ (scores)	13.22 ± 8.02	-	-
Hoehn and Yahr scale (stages)	2.65 ± 0.41	2.33 ± 0.44	0.008 ^c^
UPDRS total (scores)	53.30 ± 12.77	46.38 ± 12.35	0.043 ^b^
UPDRS Part Ⅰ (scores)	3.37 ± 1.91	2.88 ± 1.31	0.262 ^b^
UPDRS Part Ⅱ (scores)	11.94 ± 5.41	7.63 ± 4.77	0.002 ^b^
UPDRS Part Ⅲ (scores)	34.81 ± 7.30	33.90 ± 8.00	0.655 ^b^
UPDRS Part Ⅳ (scores)	3.17 ± 2.64	1.97 ± 1.88	0.105 ^c^
More affected limb (left/right)	16/11	21/9	0.420 ^a^

All data represent the means ± standard deviations; PD, Parkinson’s disease; BMI, body mass index; MMSE, Mini-Mental State Examination; LED, levodopa equivalent dose; NFOGQ, New Freezing of Gait Questionnaire; UPDRS, Unified Parkinson’s Disease Rating Scale; significant difference, *p* < 0.05; ^a^, *p*-value of Fisher’s exact test; ^b^, *p*-value of Independent *t*-test; ^c^, *p*-value of Mann-Whitney *U* test.

**Table 2:** List of variables and their definitions used for analysis.

Parameters	Variables	Description
Spatiotemporal parameter	Total steps and duration	Total steps and duration were calculated within the analysis phase.
	Step width	Step width was defined as the perpendicular distance in the lateral plane to the initial heel contact of one lower limb and the initial heel contact of the opposite lower limb (Figure 2b).
	Inner and outer step lengths	Inner and outer step lengths were defined as the perpendicular distance in the AP plane between the initial heel contact of the inner/outer lower limb and the initial heel contact of the other lower limb, respectively (Figure 2b).
	Inner and outer single support phases	Inner and outer single support phases were defined as when either the inner or outer foot was in contact with the ground (Figure 3a).
	Inner and outer double support phases	Inner and outer double support phases were defined as when both inner/outer feet were in contact with the ground (Figure 3a).
	Inner and outer stance phases	Inner and outer stance phases were defined as the inner/outer foot contacting the ground, moving from initial heel contact to toe-off (Figure 3a).
Kinematic parameter	ROM	ROM was calculated from the maximum and minimum joint (inner and outer hip, knee, ankle, shoulder, pelvis, and thorax) angles during 180° turning.
	Inner and outer toe clearance height	Inner and outer toe clearance height was selected as the maximum vertical height of the toe marker in the swing phase after toe-off for each step.
	Maximum anti-phase	Maximum anti-phase was calculated as the maximum angle (*θ*) between the lateral ASIS vector and lateral acromion processes vector in the horizontal plane during 180° turning (Figure 3b) [11].
	Incline angle	Incline angle indicated the degree of body tilt during turning and was calculated as the maximum angle (*θ*) on the lateral plane between the vector from the center of the base of support to the center of the head segment and the vertical vector of the global coordinate system located on the cone (Figure 3c) [15].
	Inner/outer ipsilateral and contralateral tempo	Inner/outer ipsilateral and contralateral tempo were calculated using the lateral humeral epicondyle and lateral femoral epicondyle markers to determine the time difference (Δt) reaching the peak position in the AP plane of the inner-inner (Δt_1_)/outer-outer (Δt_2_) (ipsilateral) and inner-outer (Δt_3_)/outer-inner (Δt_4_) (contralateral) limbs (Figure 3d) [15].
COM parameter	AP and ML RMS distances	The area of 95% CI was calculated using the trajectory of the COM on the horizontal plane during the 180° turning tasks, followed by the AP and ML RMS distances, total distance, and average speed [9].
	Total distance and average speed	

COM, center of mass; ROM, range of motion; Tempo, temporal coordination parameters of the upper and lower limbs; AP, anteroposterior; ML, mediolateral; RMS, root mean square; ASIS, anterior superior iliac spine; 95% CI, confidence interval.

**Table 3:** Stepwise binary logistic regression analysis results for freezers and non-freezers during the 180° turning task.

Variable		Cutoff Value	*β* (SE)	OR (95% CI)	*p*-Value	R_N_^2^
IMA	Outer ankle ROM	31.0°	−0.221	0.802	0.026	0.735
			(0.100)	(0.659–0.974)		

Dependent variable: 1 = freezer, 0 = non-freezer; cutoff value was determined using Youden’s index; model adjusted for age, sex, height, body mass index, disease duration, levodopa equivalent dose, Hoehn and Yahr scale, UPDRS total scores, and UPDRS Part II scores; *β*, logistic regression coefficient; SE, standard error; OR, odds ratio; 95% CI, confidence interval; R_N_^2^, Model Fit Statistics Nagelkerke; IMA, inner step of the more affected limb; ROM, range of motion; significant difference, *p* < 0.05.

**Table 4:** Association between NFOGQ scores and turning characteristics for freezers during the 180° turning task.

Variables		*β*	SE	*t*-Value	*p*-Value	VIF	Tolerance Limit
Constant		160.710	58.697	2.738	0.013		
IMA	AP RMS distance of the COM	61.272	28.589	2.143	0.045	1.405	0.712
OMA	Inner stance phase	1.027	0.399	2.571	0.018	1.390	0.720
Adjusted R^2^ = 0.501, *F* = 5.353, *p* = 0.002							

Model adjusted for age, sex, height, and body mass index; NFOGQ, New Freezing of Gait Questionnaire; IMA, inner step of the more affected limb; OMA, outer step of the more affected limb; *β*, logistic regression coefficient; SE, standard error; VIF, variance inflation factors; AP, anteroposterior; RMS, root mean square; COM, center of mass; significant difference, *p* < 0.05.

## Data Availability

The data that support the findings of this study are available from the corresponding author upon reasonable request.

## Supplementary Materials

The following supporting information can be downloaded at: https://www.mdpi.com/article/10.3390/ijerph191912131/s1, Supplementary Table S1: Results of differences between groups (freezers compared with non-freezers) and within turning directions (IMA and OMA) during the 180° turning task at the maximum speed; Supplementary Table S2: Partial correlation analysis between NFOGQ scores and all turning characteristics of freezers during the 180° turning task at the maximum speed.
