# Supplementary material for: Association between Severity of Freezing of Gait and Turning Characteristics in People with Parkinson’s Disease
Source: Int J Environ Res Public Health. 2022 Sep 25;19(19):12131. doi: 10.3390/ijerph191912131 (PMC9564463; doi:10.3390/ijerph191912131)
Supplement: Supplementary file 1 [file ijerph-19-12131-s001.zip › ijerph-1899681-supplementary.pdf]

Supplementary Table S1. Results of differences between groups (freezers compared with non-freezers) and within turning directions (IMA and OMA) during the 180° turning task at the maximum speed.

Variables		Freezers (95% CI)	Non-Freezers (95% CI)	*F*-Value	Post Hoc *p*-Value	ES
Total steps (steps)	IMA	10.93 ± 2.80 (9.92–12.06)	9.48 ± 2.15 (8.74–10.27)	2.621 (G)	**0.031**[a]	0.05
	OMA	10.14 ± 3.29 (8.88–11.42)	9.58 ± 2.65 (8.72–10.60)	1.036 (D)	0.505[b]	0.02
	Post hoc *p*-value	0.990[c]	0.149[c]	1.264 (G×D)		0.02
Total duration (s)	IMA	6.26 ± 2.21 (5.41–7.17)	5.46 ± 1.59 (4.92–6.00)	1.858 (G)	0.114[a]	0.04
	OMA	5.80 ± 2.27 (4.87–6.64)	5.46 ± 1.92 (4.84–6.23)	6.820 (D)*	0.560[a]	0.12
	Post hoc *p*-value	**0.044**[c]	0.152[c]	0.521 (G×D)		0.01
Step width (m)	IMA	0.21 ± 0.06 (0.19–0.23)	0.23 ± 0.07 (0.20–0.26)	0.537 (G)	0.263[a]	0.01
	OMA	0.24 ± 0.08 (0.21–0.27)	0.24 ± 0.08 (0.21–0.26)	2.797 (D)	0.972[a]	0.05
	Post hoc *p*-value	0.448[c]	0.213[c]	0.477 (G×D)		0.01
Inner step length (m)	IMA	0.42 ± 0.21 (0.34–0.50)	0.40 ± 0.14 (0.35–0.45)	0.040 (G)	0.637[b]	0.00
	OMA	0.34 ± 0.22 (0.26–0.42)	0.38 ± 0.16 (0.33–0.44)	8.028 (D)*	0.451[a]	0.14
	Post hoc *p*-value	0.058[c]	**0.029**[c]	1.064 (G×D)		0.02
Outer step length (m)	IMA	0.37 ± 0.16 (0.31–0.43)	0.40 ± 0.14 (0.35–0.45)	0.671 (G)	0.455[a]	0.01
	OMA	0.34 ± 0.19 (0.27–0.41)	0.37 ± 0.17 (0.31–0.44)	11.440 (D)*	0.564[a]	0.18
	Post hoc *p*-value	**0.017**[c]	**0.033**[c]	0.004 (G×D)		0.00
Inner single support phase (%)	IMA	39.80 ± 2.63 (38.83–40.83)	39.38 ± 3.24 (38.27–40.54)	0.337 (G)	0.650[a]	0.01
	OMA	39.28 ± 3.23 (38.10–40.49)	40.33 ± 2.90 (39.23–41.39)	4.009 (D)	0.183[a]	0.07
	Post hoc *p*-value	0.239[c]	0.205[c]	2.528 (G×D)		0.05
Outer single support phase (%)	IMA	40.28 ± 3.31 (39.13–41.60)	40.61 ± 3.30 (39.36–41.69)	0.309 (G)	0.723[a]	0.01
	OMA	39.88 ± 3.55	38.89 ± 3.71	0.490 (D)	0.250[a]	0.01

		(38.60–41.16)	(37.64–40.19)			
	Post hoc *p*-value	**0.020**[c]	0.410[c]	1.731 (G×D)		0.03
Inner double support phase (%)	IMA	23.85 ± 3.20 (22.63–25.10)	23.61 ± 4.31 (22.15–25.10)	0.628 (G)	0.812[a]	0.01
	OMA	24.81 ± 3.79 (23.40–26.29)	23.75 ± 4.10 (22.37–25.23)	0.012 (D)	0.278[a]	0.00
	Post hoc *p*-value	0.451[c]	0.477[c]	0.666 (G×D)		0.01
Outer double support phase (%)	IMA	24.72 ± 3.12 (23.50–25.90)	22.62 ± 4.40 (21.04–24.15)	1.348 (G)	**0.036**[a]	0.03
	OMA	24.55 ± 3.67 (23.21–25.96)	24.74 ± 4.86 (23.08–26.49)	0.017 (D)	0.849[a]	0.00
	Post hoc *p*-value	0.801[c]	0.848[c]	3.224 (G×D)		0.06
Inner stance phase (%)	IMA	63.01 ± 4.61 (61.20–64.81)	62.88 ± 3.45 (61.69–64.05)	0.027 (G)	0.949[a]	0.00
	OMA	64.27 ± 3.28 (62.97–65.51)	64.08 ± 3.43 (62.92–65.40)	0.945 (D)	0.840[a]	0.02
	Post hoc *p*-value	0.472[c]	0.764[c]	0.007 (G×D)		0.00
Outer stance phase (%)	IMA	64.40 ± 3.36 (63.13–65.75)	63.06 ± 3.79 (61.69–64.37)	2.594 (G)	0.138[a]	0.05
	OMA	64.43 ± 3.71 (63.08–65.82)	63.50 ± 3.93 (62.12–65.04)	0.188 (D)	0.305[a]	0.00
	Post hoc *p*-value	0.234[c]	0.594[c]	0.115 (G×D)		0.00
Inner hip ROM (°)	IMA	51.38 ± 18.43 (44.49–58.28)	55.61 ± 18.37 (48.93–62.20)	3.598 (G)	0.361[a]	0.07
	OMA	46.76 ± 14.86 (41.40–52.70)	56.44 ± 18.85 (50.19–63.12)	1.629 (D)	**0.049**[b]	0.03
	Post hoc *p*-value	0.338[c]	0.516[c]	0.891 (G×D)		0.02
Outer hip ROM (°)	IMA	51.96 ± 17.94 (45.42–58.85)	55.04 ± 18.09 (48.33–61.68)	2.654 (G)	0.512[a]	0.05
	OMA	46.56 ±15.56 (41.21–52.51)	55.73 ± 18.62 (49.78–62.66)	0.684 (D)	0.054[a]	0.01
	Post hoc *p*-value	0.729[c]	0.582[c]	1.238 (G×D)		0.02
Inner knee ROM (°)	IMA	58.89 ± 13.97 (53.94–64.15)	62.21 ± 16.26 (56.19–68.13)	1.605 (G)	0.300[a]	0.03
	OMA	57.67 ± 14.24 (52.69–63.30)	60.64 ± 12.76 (56.16–65.09)	0.738 (D)	0.345[a]	0.01

	Post hoc *p*-value	0.916[c]	0.337[c]	0.015 (G×D)		0.00
	IMA	58.69 ± 13.59 (53.74–63.93)	62.05 ± 15.53 (56.45–67.51)	1.476 (G)	0.299[a]	0.03
Outer knee ROM (°)	OMA	58.57 ± 15.70 (52.65–64.43)	61.47 ± 13.11 (56.52–65.83)	1.359 (D)	0.391[a]	0.03
	Post hoc *p*-value	0.485[c]	0.070[c]	0.020 (G×D)		0.00
	IMA	30.02 ± 10.63 (26.29–34.34)	33.76 ± 10.43 (29.94–38.05)	1.606 (G)	0.167[a]	0.03
Inner ankle ROM (°)	OMA	29.99 ± 7.87 (27.09–33.07)	31.07 ± 8.28 (28.20–33.92)	0.098 (D)	0.511[a]	0.00
	Post hoc *p*-value	0.759[c]	0.729[c]	1.041 (G×D)		0.02
	IMA	28.79 ± 8.05 (25.93–32.03)	33.13 ± 9.13 (29.56–36.44)	3.900 (G)	**0.048**[a]	0.07
Outer ankle ROM (°)	OMA	29.58 ± 7.81 (26.74–32.50)	32.87 ± 10.35 (29.43–36.47)	0.298 (D)	0.161[a]	0.01
	Post hoc *p*-value	0.082[c]	0.536[c]	0.317 (G×D)		0.01
	IMA	6.78 ± 1.31 (6.29–7.32)	7.11 ± 0.94 (6.80–7.46)	3.572 (G)	0.108[a]	0.07
Inner toe clearance height (cm)	OMA	6.70 ± 1.38 (6.18–7.20)	7.15 ± 1.03 (6.80–7.51)	0.481 (D)	0.076[a]	0.01
	Post hoc *p*-value	0.834[c]	0.501[c]	0.358 (G×D)		0.01
	IMA	6.64 ± 1.16 (6.20–7.08)	6.86 ± 1.20 (6.47–7.31)	1.846 (G)	0.334[a]	0.04
Outer toe clearance height (cm)	OMA	6.65 ± 1.16 (6.23–7.09)	6.99 ± 0.99 (6.67–7.33)	0.229 (D)	0.136[a]	0.00
	Post hoc *p*-value	0.318[c]	0.964[c]	0.331 (G×D)		0.01
	IMA	30.35 ± 12.26 (26.01–35.01)	36.56 ± 14.57 (31.80–42.14)	2.700 (G)	0.084[a]	0.05
Inner shoulder ROM (°)	OMA	31.70 ± 12.38 (27.36–36.37)	36.80 ± 18.04 (30.93–43.27)	5.983 (D)*	0.207[a]	0.11
	Post hoc *p*-value	0.247[c]	**0.030**[c]	0.133 (G×D)		0.00
	IMA	31.72 ± 13.98 (26.62–36.88)	35.20 ± 14.17 (30.13–40.25)	0.225 (G)	0.326[a]	0.00
Outer shoulder ROM (°)	OMA	34.55 ± 14.52 (29.36–39.71)	33.79 ± 14.97 (28.34–39.13)	2.583 (D)	0.885[a]	0.05
	Post hoc	0.426[c]	0.099[c]	1.677 (G×D)		0.03

	p-value					
Pelvis ROM (°)	IMA	32.61 ± 18.39 (26.29–39.90)	30.94 ± 15.93 (25.09–36.78)	0.270 (G)	0.879[b]	0.01
	OMA	32.98 ± 16.32 (26.91–39.26)	30.11 ± 16.51 (24.72–36.61)	0.007 (D)	0.492[b]	0.00
	Post hoc *p*-value	0.737[d]	0.704[d]	0.075 (G×D)		0.00
Thorax ROM (°)	IMA	39.16 ± 18.04 (32.69–46.29)	31.52 ± 16.98 (25.42–38.12)	3.969 (G)	0.071[b]	0.07
	OMA	39.83 ± 16.61 (33.87–46.78)	32.28 ± 14.07 (27.67–37.79)	0.067 (D)	0.075[a]	0.00
	Post hoc *p*-value	0.096[c]	0.261[c]	0.001 (G×D)		0.00
Maximum anti-phase (°)	IMA	18.33 ± 7.87 (15.53–21.51)	22.32 ± 8.06 (19.61–25.34)	2.434 (G)	0.081[a]	0.05
	OMA	18.66 ± 8.00 (15.92–21.75)	21.29 ± 9.09 (18.35–24.84)	0.015 (D)	0.286[a]	0.00
	Post hoc *p*-value	0.579[c]	0.746[c]	0.443 (G×D)		0.01
Incline angle (°)	IMA	10.35 ± 2.94 (9.27–11.50)	10.70 ± 2.87 (9.70–11.75)	0.241 (G)	0.705[a]	0.01
	OMA	10.53 ± 3.45 (9.23–11.81)	10.91 ± 2.54 (10.00–11.84)	0.614 (D)	0.642[a]	0.01
	Post hoc *p*-value	0.999[c]	0.423[c]	0.009 (G×D)		0.00
Inner ipsilateral tempo (s)	IMA	0.18 ± 0.11 (0.14–0.23)	0.16 ± 0.09 (0.13–0.19)	1.112 (G)	0.358[a]	0.02
	OMA	0.20 ± 0.13 (0.15–0.26)	0.19 ± 0.11 (0.15–0.23)	2.822 (D)	0.574[a]	0.05
	Post hoc *p*-value	0.628[c]	0.139[c]	0.021 (G×D)		0.00
Outer ipsilateral tempo (s)	IMA	0.20 ± 0.14 (0.16–0.25)	0.20 ± 0.13 (0.16–0.25)	0.280 (G)	0.968[b]	0.01
	OMA	0.20 ± 0.12 (0.15–0.24)	0.18 ± 0.10 (0.14–0.21)	2.212 (D)	0.713[b]	0.04
	Post hoc *p*-value	0.703[d]	0.571[d]	0.136 (G×D)		0.00
Inner contralateral tempo (s)	IMA	0.50 ± 0.18 (0.43–0.57)	0.41 ± 0.22 (0.33–0.49)	3.574 (G)	0.080[a]	0.07
	OMA	0.54 ± 0.29 (0.43–0.65)	0.48 ± 0.14 (0.43–0.53)	0.569 (D)	0.286[a]	0.01
	Post hoc *p*-value	0.193[c]	0.713[c]	0.233 (G×D)		0.01

Outer contralateral tempo (s)	IMA	0.51 ± 0.32 (0.39–0.63)	0.48 ± 0.21 (0.41–0.56)	2.088 (G)	0.968[b]	0.04
	OMA	0.53 ± 0.31 (0.42–0.65)	0.41 ± 0.23 (0.33–0.49)	0.689 (D)	0.110[b]	0.01
	Post hoc *p*-value	1.000[d]	0.139[d]	1.264 (G×D)		0.02
AP RMS distance of the COM (m)	IMA	0.13 ± 0.05 (0.11–0.15)	0.13 ± 0.04 (0.12–0.15)	0.162 (G)	0.721[a]	0.00
	OMA	0.14 ± 0.05 (0.12–0.16)	0.13 ± 0.05 (0.11–0.14)	0.345 (D)	0.371[a]	0.01
	Post hoc *p*-value	0.583[c]	0.889[c]	1.052 (G×D)		0.02
ML RMS distance of the COM (m)	IMA	0.56 ± 0.22 (0.48–0.64)	0.53 ± 0.21 (0.45–0.60)	0.185 (G)	0.437[a]	0.00
	OMA	0.53 ± 0.24 (0.44–0.62)	0.54 ± 0.19 (0.47–0.62)	0.541 (D)	0.936[b]	0.01
	Post hoc *p*-value	0.766[c]	0.553[c]	0.443 (G×D)		0.01
Total distance of the COM (m)	IMA	2.36 ± 0.57 (2.16–2.58)	2.23 ± 0.69 (1.99–2.48)	1.263 (G)	0.473[a]	0.02
	OMA	2.45 ± 0.75 (2.16–2.71)	2.24 ± 0.64 (1.98–2.47)	0.210 (D)	0.160[b]	0.00
	Post hoc *p*-value	0.358[c]	0.269[c]	0.136 (G×D)		0.00
Average speed of the COM (m/s)	IMA	0.41 ± 0.18 (0.35–0.48)	0.42 ± 0.18 (0.36–0.48)	0.103 (G)	0.749[b]	0.00
	OMA	0.48 ± 0.26 (0.39–0.59)	0.44 ± 0.16 (0.39–0.49)	1.779 (D)	0.905[b]	0.03
	Post hoc *p*-value	0.141[d]	0.475[d]	0.617 (G×D)		0.01

All data represent the means ± standard deviations; 95% CI, confidence interval; ES, effect size; IMA, inner step of the more affected limb; OMA, outer step of the more affected limb; ROM, range of motion; Tempo, temporal coordination parameters of the upper and lower limbs; AP, anteroposterior; ML, mediolateral; RMS, root mean square; COM, center of mass; adjusting for age, sex, height, and body mass index; G, main effects between groups; D, main effects within turning directions; G×D, interaction effects between group and turning direction; *, $p < 0.05$; boldface denotes a significant difference ($p < 0.05$).

[a] *p*-value of analysis of covariance (ANCOVA) between freezers and non-freezers.

[b] *p*-value of Mann-Whitney *U* test between freezers and non-freezers.

[c] *p*-value of ANCOVA between IMA and OMA.

[d] *p*-value of Wilcoxon-signed rank test between IMA and OMA.

Supplementary Table S2. Partial correlation analysis between NFOGQ scores and all turning characteristics of freezers during the 180° turning task at the maximum speed.

**Variables**	**NFOGQ**

IMA	Inner toe clearance height	−0.468 *
	AP RMS distance of the COM	0.436 *
OMA	Inner stance phase	0.502 *

Adjusting for age, sex, height, and body mass index; NFOGQ, New Freezing of Gait Questionnaire; IMA, inner step of the more affected limb; OMA, outer step of the more affected limb; AP, anteroposterior; RMS, root mean square; COM, center of mass; * $p < 0.05$.
